# May chest pain describe coronary heart disease?

**DOI:** 10.3325/cmj.2013.54.411

**Published:** 2013-08

**Authors:** Sevket Balta, Mustafa Cakar, Sait Demırkol, Ugur Kucuk, Seyit Ahmet Ay, Murat Unlu

**Affiliations:** 1 Department of Internal Medicine, Gulhane Medical Academy, Ankara, Turkey; 2 Department of Cardiology, Gulhane Medical Academy, Ankara, Turkey

To the Editor,

We read the article ‘‘Does the patient with chest pain have a coronary heart disease? Diagnostic value of single symptoms and signs – a meta-analysis” by Haasenritter et al with interest (1). The authors performed a comprehensive systematic review and quantitative meta-analysis to determine the diagnostic value of medical history and physical examination for coronary heart disease (CHD) in patients with chest pain. In addition, they explored the amount and potential sources of heterogeneity between studies. We believe that these findings will act as a guide for further studies that will assess single symptoms as a diagnostic test of CHD.

Cardiovascular diseases are a major health and economic problem and have accounted for more than 50% of all-cause mortality in the last two decades (2). Chest pain, a common symptom of CHD, is a frequent complaint in all health care settings. Chest pain may be caused by a wide range of different illnesses, among which life threatening cardiac disease is of the greatest concern. However, chest pain is caused by CHD in only around 12%-15% of primary care patients (3). Despite advanced diagnostic technology available to characterize CHD, important first steps in the evaluation of patients with chest pain are history and physical examination. Most helpful for the diagnosis of myocardial ischemia is generally the presence of typical angina, radiation of pain to the right arm/shoulder, reproducible pain by palpation, and pain that is related to breathing.

The authors found that the accuracy of several index tests varied across subgroups determined by case definition of CHD. In respect to the case definition, most useful diagnostic parameters were history of CHD, known myocardial infarction (MI), typical angina, history of diabetes mellitus, exertional pain, history of angina pectoris, and male sex for stable CHD; and radiating pain to right arm/shoulder and palpitation for MI. However, the history of CHD and known MI were the most useful parameters for diagnosis of acute coronary syndrome.

Some patients present with less-typical symptoms, such as nausea/vomiting, shortness of breath, fatigue, palpitations, or syncope. These patients tend to present later, are more likely to be women, diabetic or elderly patients, and less frequently receive reperfusion therapy and other evidence-based therapies than patients with a typical chest pain presentation. Registries show that up to 30% of patients with ST-elevation myocardial infarction present with atypical symptoms (4).

Finally, we think that typical chest pain may describe acute coronary syndrome, but the physicians should evaluate patients together with other diagnostic criteria such as symptoms, laboratory parameters, and diagnostic methods.
